# Classification of Cowpox Viruses into Several Distinct Clades and Identification of a Novel Lineage

**DOI:** 10.3390/v9060142

**Published:** 2017-06-10

**Authors:** Annika Franke, Florian Pfaff, Maria Jenckel, Bernd Hoffmann, Dirk Höper, Markus Antwerpen, Hermann Meyer, Martin Beer, Donata Hoffmann

**Affiliations:** 1 Institute of Diagnostic Virology, Friedrich-Loeffler-Institut, Federal Research Institute for Animal Health, Südufer 10, 17493 Greifswald, Germany; Annika.Franke@fli.de (A.F.); Florian.Pfaff@fli.de (F.P.); Maria.Jenckel@fli.de (M.J.); Bernd.Hoffmann@fli.de (B.H.); Dirk.Hoeper@fli.de (D.H.); Donata.Hoffmann@fli.de (D.H.); 2 Bundeswehr Institute of Microbiology, Neuherbergstr 11, 80937 Munich, Germany; markusantwerpen@bundeswehr.org (M.A.); hermann1meyer@bundeswehr.org (H.M.)

**Keywords:** cowpox virus, *Orthopoxvirus*, poxvirus, recombination, phylogeny, genetic diversity, Germany

## Abstract

Cowpox virus (CPXV) was considered as uniform species within the genus *Orthopoxvirus* (OPV). Previous phylogenetic analysis indicated that CPXV is polyphyletic and isolates may cluster into different clades with two of these clades showing genetic similarities to either variola (VARV) or vaccinia viruses (VACV). Further analyses were initiated to assess both the genetic diversity and the evolutionary background of circulating CPXVs. Here we report the full-length sequences of 20 CPXV strains isolated from different animal species and humans in Germany. A phylogenetic analysis of altogether 83 full-length OPV genomes confirmed the polyphyletic character of the species CPXV and suggested at least four different clades. The German isolates from this study mainly clustered into two CPXV-like clades, and VARV- and VACV-like strains were not observed. A single strain, isolated from a cotton-top tamarin, clustered distantly from all other CPXVs and might represent a novel and unique evolutionary lineage. The classification of CPXV strains into clades roughly followed their geographic origin, with the highest clade diversity so far observed for Germany. Furthermore, we found evidence for recombination between OPV clades without significant disruption of the observed clustering. In conclusion, this analysis markedly expands the number of available CPXV full-length sequences and confirms the co-circulation of several CPXV clades in Germany, and provides the first data about a new evolutionary CPXV lineage.

## 1. Introduction

The species cowpox virus (CPXV) is a genetically diverse and polyphyletic member of the genus *Orthopoxvirus* (OPV) in the family *Poxviridae* [1]. CPXV is assumed to be the causative agent of cow pox, a zoonotic disease that causes lesions on the udder of dairy cows and the hands of dairymaids. Whether Edward Jenner’s effective cross-protective vaccine against human smallpox (variola virus (VARV)) based on a virus belonging to the species CPXV or not is obscure. Later the used protective agent was referred to as vaccinia virus (VACV), whose natural origin has not yet been identified [2]. Differences in the phenotype of lesions on the chorioallantoic membrane of embryonated chicken eggs and the presence or absence of inclusion bodies characterized CPXV and VACV as dissimilar viruses and led to the assignment as species [3] which was later confirmed by restriction fragment length polymorphism (RFLP) analysis [4]. Sero-surveys showed, that CPXV is endemic in Western Eurasia, and wild rodents, primarily voles, are the reservoir host species [5]. However, the observed biological and experimental host range of CPXV seems to be very broad [6] and spill-over infections to accidental hosts (e.g., rats, cats, cattle, horses, lamas, zoo animals, and humans) are reported regularly, with increasing case numbers for Europe [7]. Recent confirmed zoonotic transmissions of CPXV were mainly caused by direct contact with infected pet rats [8,9], cats [10,11], or zoo animals [12,13,14]. Although human infections are often mild and self-limiting, immunocompromised patients can develop a systemic and fatal outcome of disease [15,16,17,18].

Compared to all other known members of the genus OPV, CPXVs have the largest genome (above 220 kbp) and the most extensive genetic repertoire [19,20]. The central region of the genome is highly conserved and contains genes involved in key functions, such as replication, transcription, and virion-assembly. In contrast, genes located in the terminal genomic regions encode proteins involved in the interaction with the host in order to reduce their anti-viral processes. Therefore, these genes have been described as “virulence genes” [21].

The differentiation of CPXV from other OPV species, such as VACV, monkeypox virus (MPXV), and VARV, was commonly based on phenotypic features like lesions, types of cellular inclusion bodies [22], and RFLP pattern [19]. Nowadays, PCR-based approaches and sequencing of partial genes allows a much more sensitive and robust differentiation for routine diagnostics [23,24]. The advent of high-throughput sequencing approaches resulted in a growing number of available full-length OPV genomes, and phylogenetic analysis indicated, that CPXV is a diverse and polyphyletic group [25,26,27]. The historically-based unity of CPXV is, therefore, currently under revision and more data from circulating strains is urgently needed. 

In order to gain further insights into the evolutionary diversity of CPXV, we determined full-length sequences of 20 CPXV strains that have been isolated over the last seven years from animals and humans in Germany. The provided data and analyses may be used for further re-classification of the genetically versatile species of CPXV.

## 2. Materials and Methods

### 2.1. Selection and Isolation of CPXV Strains

During surveillance of the German National Reference Laboratory for Monkeypox (situated within the Friedrich-Loeffler-Institut, Isle of Riems, Germany) several animal-derived samples (taken for routine diagnostics) were subject to CPXV diagnostics. Briefly, DNA was extracted from various organ tissues using the QIAamp DNA Mini Kit (Qiagen, Hilden, Germany) and OPV specific DNA was detected using a quantitative polymerase chain reaction (qPCR) as described elsewhere [28]. Organ material that scored positive in the qPCR was propagated on Vero76 cells (Collection of Cell Lines in Veterinary Medicine CCLV, Friedrich-Loeffler-Institut). Routine diagnostic analysis at the Bundeswehr Institute of Microbiology led to isolation of four strains: CPXV strain Ger/2007/Vole was derived from the lung tissue of a common vole (*Microtus arvalis*) which had been trapped on a military training ground close to Rottweil (Germany) during a survey to assess the zoonotic potential of rodents. CPXV strains Ger/2015/Human1 and Ger/2015/Human2 were isolated from local lesions on the neck of a veterinary assistant and the head of a farmer, respectively (all samples were taken during routine diagnostics of these cases). In all three cases, virus isolation, genomic DNA extraction and identification as CPXV was performed, as already described for isolate Ger/2014/Human [29]. A summary of isolates used in this study, along with information about the sampling place, year, host, and clinical description can be found in Table 1. Case reports and partial sequences have been described for isolates Ger 2010 MKY [30] and Ger/2014/Human [29].

### 2.2. High-Throughput Sequencing

Full-length sequencing of CPXV isolates was conducted as described earlier [27]. In brief, DNA was extracted from infected cell cultures using the High Pure PCR Template Preparation Kit (Roche, Mannheim, Germany) and 0.5–1 μg of DNA was fragmented (mean of 300 bp) using the Covaris M220 ultrasonicator (Covaris, Brighton, UK). Illumina-compatible sequencing libraries were prepared using NEXTflex DNA barcodes (Bioo Scientific, Austin, TX, USA) and SPRIworks Fragment Library Cartridge II (Beckman Coulter, Fullerton, CA, USA) on a SPRI-TE library system (Beckman Coulter). Size exclusion of the library was done manually using Ampure XP magnetic beads (Beckman Coulter) and was controlled on a Bioanalyzer 2100 (Agilent Technologies, Böblingen, Germany) using a high-sensitivity DNA chip and corresponding reagents. A Kapa Library Quantification Kit (Kapa Biosystems, Wilmington, DE, USA) was further used for quantification of the final libraries. Sequencing was performed on an Illumina MiSeq using MiSeq reagent kit, version 2 and version 3 (Illumina, San Diego, CA, USA).

### 2.3. De Novo Assembly and Genome Annotation

Raw reads were quality trimmed and assembled de novo using the 454 Sequencing System Software (v. 2.8; Roche, Mannheim, Germany), and the resulting contigs were arranged in order to match the CPXV genome. Draft CPXV genomes were further confirmed by reference guided mapping (454 Sequencing System Software) using the “–rst 0” parameter with respect to their repetitive genomic termini. The mean genomic coverage of each full-length CPXV sequence exceeded the minimal acceptable coverage of 20. Full-length CPXV sequences were annotated analogue to the nomenclature of the CPXV Brighton Red reference strain (AF482758) as described elsewhere [27].

### 2.4. Accession Numbers

Annotated full-length CPXV sequences were uploaded to the European Nucleotide Archive (ENA) and made publicly available under the study accession PRJEB20974.

### 2.5. Phylogenetic Analysis

A total of 83 full-length OPV sequences, including 63 publicly available and 20 novel sequences from this study, were selected for the phylogenetic analysis. The dataset comprised representative sequences from Old World OPV species camelpox virus (CMLV), CPXV, ectromelia virus (ECTV), MPXV, taterapox virus, VACV, and VARV, as well as New World OPV species, racoonpox virus, skunkpox virus, and volepox virus [1]. Details of the strains used, including accession numbers, can be found in the supplementary material (Table S1). The sequences were initially aligned using the MAFFT plugin (version 7.222; [31]) as incorporated in the Geneious software (version 10.0.9; Biomatters Inc., Aukland, New Zealand, [32]). In order to remove badly aligned or putatively non-homologous regions from the alignment we used the gBlocks program (version 0.91b; [33]) utilizing a minimum block length of five. Subsequently, maximum-likelihood (ML) phylogeny was inferred using IQ-TREE (multicore version 1.5.4; [34]) with options for optimal model selection, considering FreeRate heterogeneity (TVM+R4), and 100,000 ultra-fast bootstrap replicates [35]. Trees were visualized in FigTree (version 1.4.0). Inter- and intra-clade distances were calculated using the uncorrected p-distance with pairwise deletion, as incorporated in MEGA (version 7.0.18; [36]).

Alterations in tree topologies were analyzed by splitting the aforementioned alignment into 28 smaller segments of each 5000 nt and a single segment of 2286 nt. Phylogenetic trees were calculated for each segment as described above and supported by 1000 ultra-fast bootstrap replicates. Based on these trees we subsequently calculated a consensus network [37] using median edge weights and a threshold of 10% as incorporated in SplitsTree4 (version 4.13.1; [38]). In order to further address potential recombination events, a bootscan analysis [39] using the RDP4 program (version 4.85; [40]) was conducted. In detail, potential recombinant sequences were scanned against appropriate reference sequences from each OPV clade over the aforementioned alignment using the Jukes-Cantor substitution model, a sliding window of 5000 nt, a step size of 100 nt and bootstrap support by 100 replicates. The bootstrap cut-off was set to 70%.

### 2.6. Geographic Analysis

For geographic analysis, the sampling places of 58 (38 public, 20 new) CPXV strains, selected from the aforementioned 83 full-length OPV, were plotted to a map, using ArcGIS Software (version 10.2.2; ESRI, Redlands, CA, USA). A summary of strains used and coordinates can be found in the Supplemental Table S2.

## 3. Results

### 3.1. Novel CPXV Isolates

**Figure 1:**
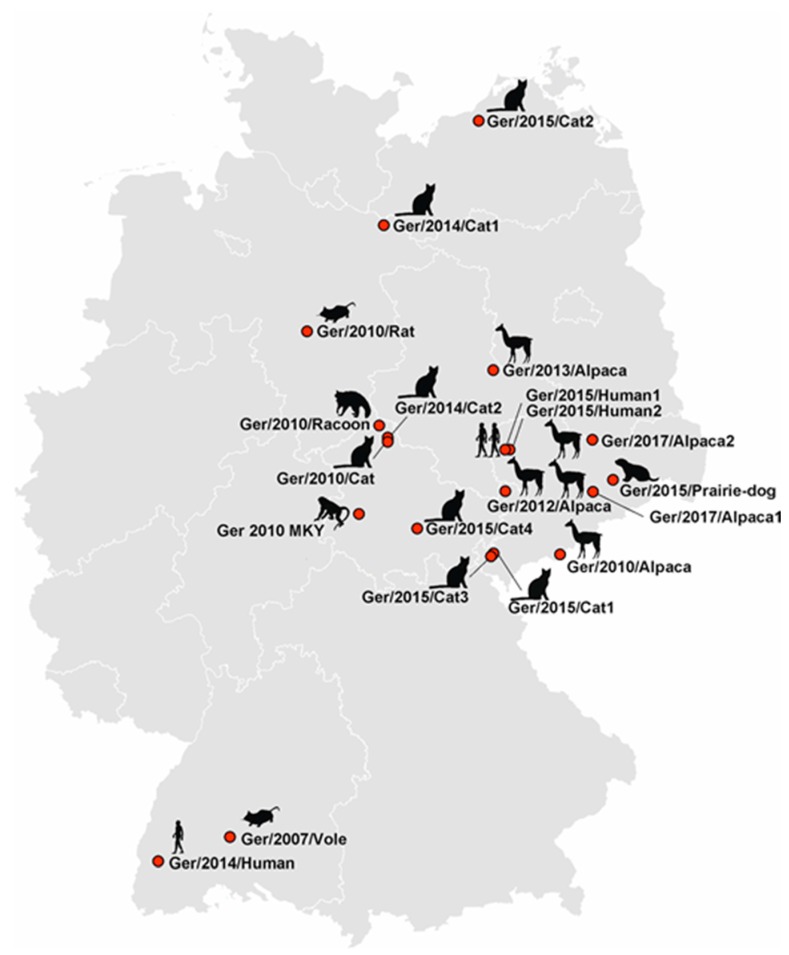
Origin of 20 cowpox virus strains from Germany. Hosts are depicted as black silhouettes accompanied by strain designation.

During the years 2010–2017, the German National Reference Laboratory for Monkeypox confirmed a total of 28 CPXV-positive animal cases. From these, 16 CPXV strains could be isolated in Vero76 cell cultures using homogenates of organs of seven cats, five alpacas, a rat, a cotton-top tamarin, a raccoon, and a prairie dog (Table 1). In addition, one isolate was obtained directly from a common vole (*Microtus arvalis*) sampled during rodent screening by the Bundeswehr Institute of Microbiology. Three additional isolates were derived from epidemiologically-independent human cases in Germany. The sampling places of these 20 isolates were mainly located in the Eastern and Southern parts of Germany (Figure 1). From all isolates the full-length genome sequence was determined.

### 3.2. CPXV Phylogeny

**Figure 2:**
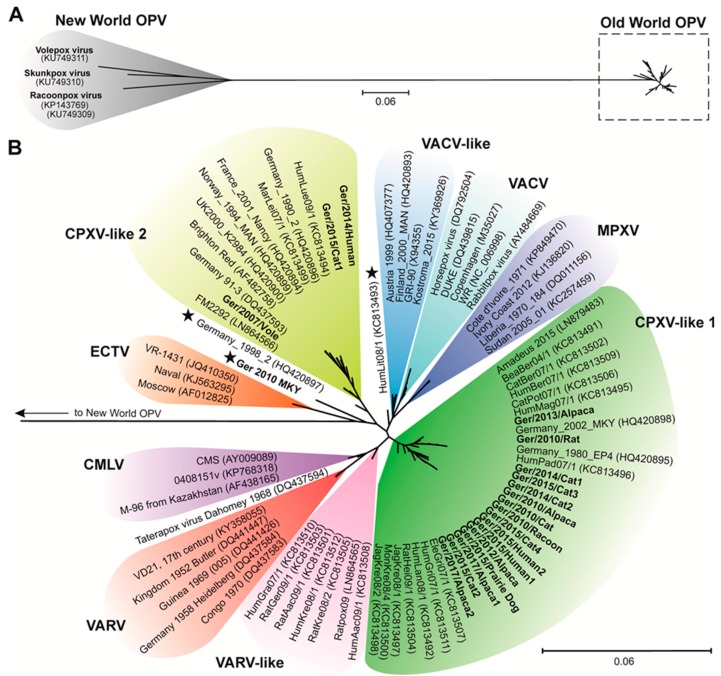
Phylogeny of Orthopoxviruses (OPVs). (**A**) New-World (highlighted grey) and Old-World OPVs (dotted box) were clearly separated by the unrooted phylogenetic tree. (**B**) Enlarged unrooted phylogenetic tree of Old-World OPVs defined the species ectromelia virus (ECTV), monkeypox virus (MPXV), vaccinia virus (VACV), and variola virus (VARV) as monophyletic clades. Cowpox virus (CPXV) strains were polyphyletic and grouped into four different clades (CPXV-like 1, CPXV-like 2, VACV-like, VARV-like). Single-branch CPXV isolates are indicated by a black asterisk. Isolates sequenced in this study appear in bold face. Scales represent substitutions per position. All species and clade segregating branches are supported by bootstrap values of at least 80% (see Supplementary Figure S1).

Together with representative sequences received from public nucleotide archives, a total of 83 full-length OPV sequences were aligned. Discarding nucleotide positions not present in all strains 142,286 nt were used for analyzing the phylogenetic relationship. The resulting phylogenetic tree clearly separated New- and Old-World OPV species into two sister groups (Figure 2A). The phylogenetic distance between both groups (15.4%) was rather high in comparison to the distance observed within them (New-World OPV species: 8.3%, Old-World OPV species: 2.0%), respectively. Regarding the Old-World OPV group, virus strains belonging to the species ECTV, MPXV, VACV, VARV, and CMLV clearly formed distinct clades (Figure 2B). Taterapox (TATV) appeared as a single branch. In contrast, CPXV strains were polyphyletic and did not form a single phylogenetic group. We identified in our analysis four different CPXV clades, which we tentatively named CPXV-like 1, CPXV-like 2, VARV-like, and VACV-like clades. Three of the novel CPXV isolates described here grouped into the CPXV-like 2 clade (15%), and 16 belonged to the CPXV-like 1 clade (80%). The strains CPXV HumLit08/1, CPXV Germany_1998_2, and CPXV Ger 2010 MKY appeared as single branches (Figure 2B, indicated by an asterisk). CPXV Ger 2010 MKY from a cotton-top tamarin is separated from all other clades, and its closest phylogenetic neighbors are virus strains belonging to the species ECTV. We did not observe any relation between the hosts of the analyzed CPXV strains and their phylogenetic clustering into the clades. Interestingly, the common vole isolate CPXV Ger/2007/Vole was grouped together with CPXV FM2292, another CPXV strain originating from a common vole. The overall nucleotide sequence identity of both vole isolates was about 99% based on the described alignment.

### 3.3. CPXV Consensus Network and Bootscan Analysis

**Figure 3:**
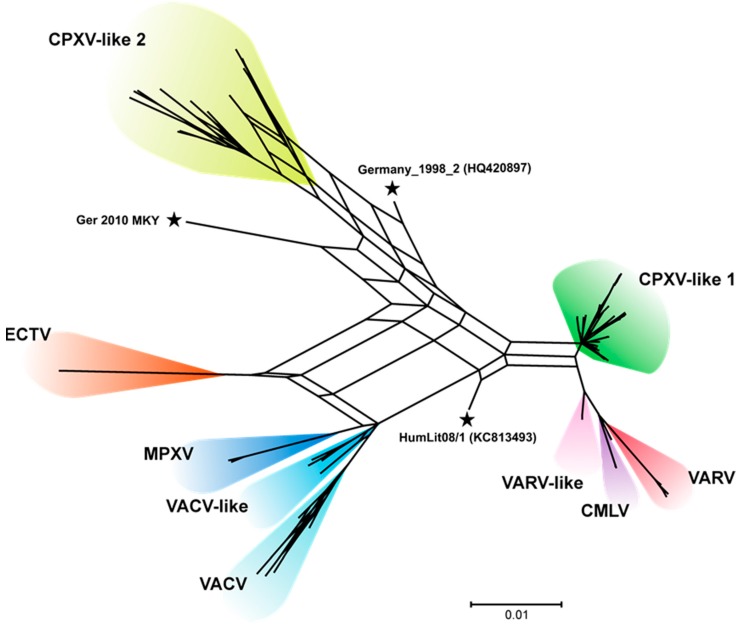
A consensus network calculated from 29 sub-genomic segments of the Orthopoxviruses (OPV) alignment indicates recombination between the clades. The genome alignment of 83 OPV was split into 28 segments of each 5000 nt and a single segment of 2286 nt and phylogenetic trees were constructed for each segment. The trees were further combined into a consensus network with SplitsTree software, visualizing incompatibilities between them. Splits that are conserved within all trees will produce unique bifurcating trees (coloured), while splits that are present in only some of the trees (at least 10%) will result in box-like structures. Box-like structures are therefore created by strains that consist of genomic segments that cluster into different genetic groups. The variable grouping of genomic segments in a single strain might be interpreted as a result of recombination. The established OPV clades were clearly separated, while isolates that appeared as single branches in the conventional phylogenetic analyses (black asterisks) were again grouped between them. Box-like structures between the clades possibly indicate recombination events during their evolution. The scale represents substitutions per position and the New-World OPVs are hidden in the illustration.

A phylogenetic consensus network from the 83 sequences was created to analyze the three CPXV strains appearing as single branch in the phylogenetic tree in more detail. This consensus network is a combination of 29 phylogenetic trees showing incompatibilities between them. The defined four CPXV clades were confirmed and clearly distinguishable from each other. The CPXV strains HumLit08/1, CPXV Germany_1998_2, and CPXV Ger 2010 MKY again appeared as single branches. Nevertheless, the CPXV isolate Germany_1998_2 contained parts from CPXV-like 1 and the CPXV-like 2 clade showed by the edges of the network. In contrast, the CPXV isolate HumLit08/1 showed edges of the network from the CPXV-clade 1, VARV-like and VACV-like, whereas the new CPXV strain Ger 2010 MKY seemed to be positioned separately (Figure 3).

**Figure 4:**
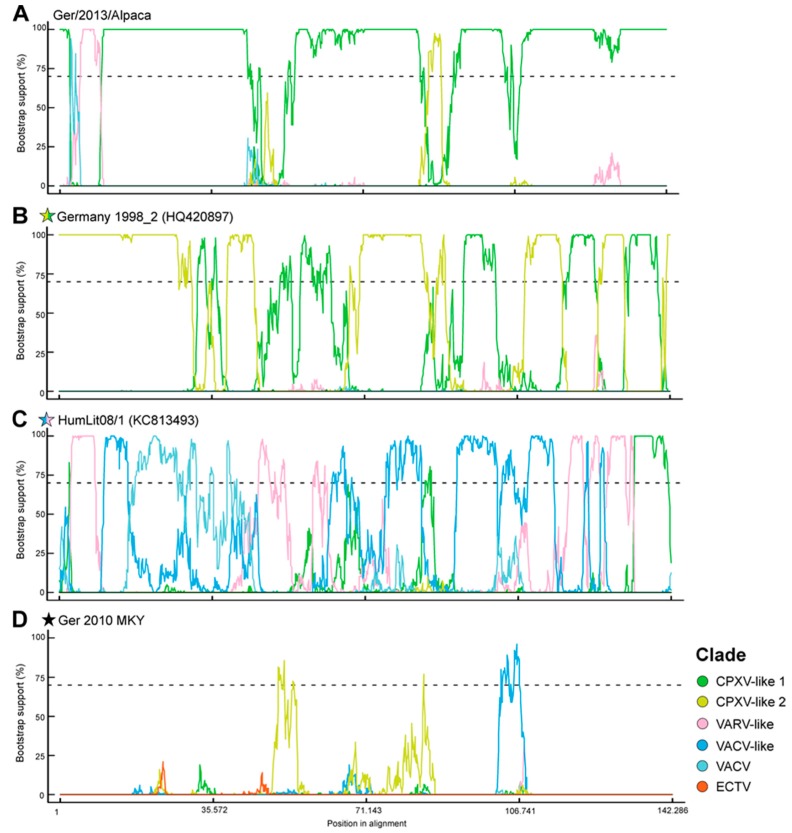
Bootscan analysis performed with RDP4 software reveals potential recombination events between cowpox virus (CPXV) clades. (**A**) The exemplary chosen CPXV isolate Ger/2013/Alpaca (CPXV-like 1) shows only minor influences of recombination. (**B**) Genomic regions of Germany 1998_2 cluster either within the CPXV-like 1 or 2 clade. (**C**) A more complex mosaic-like pattern was observed for the sequence of HumLit08/1 that comprises genomic segments related to VACV-like, VACV, and VARV-like clades. (**D**) In contrast to that, the CPXV isolate Ger 2010 MKY is phylogenetically distant from all of the established clades, resulting in only a few significant groupings.

These edges, which may be indicators of recombination events, were further analysed by a so-called bootscan analysis. As already indicated by the consensus network, the CPXV strain Germany 1998_2 showed genomic regions which either clustered in the CPXV-like 1 or the CPXV-like 2 clade. The genomic sequence of the CPXV isolate HumLit08/1 grouped within the VACV- or the VARV-like clade. The new strain CPXV Ger 2010 MKY in contrast showed less recombination and can, therefore, be regarded as a CPXV strain establishing a novel CPXV lineage separated from the defined CPXV clades (Figure 4).

### 3.4. CPXV Geographic Distribution

**Figure 5:**
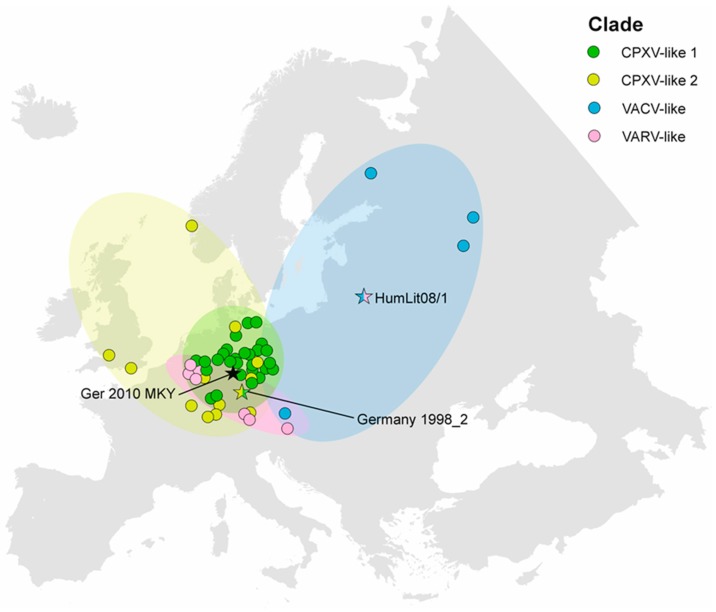
Sampling places of cowpox virus (CPXV) strains from different phylogenetic clades in Europe. The clade CPXV-like 2 seems to be restricted to the Northern and Western parts of Europe, while VACV-like strains are present in Eastern Europe. CPXV-like 1 has so far only been observed within Germany. In Central Europe all phylogenetic CPXV clades, including the VARV-like clade and a putative new clade that is represented by isolate Ger 2010 MKY (black asterik), seems to be co-circulating. The potential recombinant isolates Germany 1998_2 and HumLit08/1 are highlighted by multicolored asteriks according to their genomic composition.

The sampling places of all CPXV strains included in this study (37 publicly available and 20 strains described here) were plotted onto a map of Europe in order to analyse a potential correlation to their phylogeny (Figure 5). The CPXV isolates mainly originated from Germany and the neighbouring countries, Austria and France, as well as Great Britain, Norway, Finland, Lithuania, and Russia. In Central Europe, the observed CPXV isolates were present in all defined phylogenetic clades. In contrast, CPXV strains from Great Britain and Norway only grouped within the CPXV-like 1 clade. Up to now, the CPXV-like 2 clade seems to be restricted to Germany. The VACV-like clade was limited to far eastern parts of Europe and to a single case in Austria. Single-branch CPXV strains that did not cluster in any of the defined clades were found in Germany and Lithuania. Therefore, co-circulation of at least three different CPXV clades within a geographic region was verified, as well as the occurrence of viruses with mixed sequences as a result of recombination events.

## 4. Discussion

CPXV is considered as a potential re-emerging zoonotic pathogen in Europe and the annual number of human cases might be rising because the number of immunised humans (against VARV) decreases [41]. To date, numerous CPXV infections of multiple different host species have been recognized all over Western Eurasia, however, less is known about the genetic diversity of circulating clades. Previous studies showed that the species CPXV is indeed a relatively diverse group within the genus OPV and therefore seems to exist in different phylogenetic clusters [20,25,26]. Although the full spectrum of variability is only accessible by full-length genome sequences, the number of them is currently limited. In order to expand the available data, we isolated CPXV strains from different animal species, as well as humans, from Germany and determined their full-length genome sequences by high-throughput sequencing.

### 4.1. Phylogenetic Analysis

An alignment comprising most of the core genome region was used for phylogenetic and recombination analysis. Our results confirmed that the species CPXV is polyphyletic. There are two clades, which are clearly related to either VARV (VARV-like clade) or VACV (VACV-like clade), in accordance to previous analysis [20,25,26]. In addition, we confirmed two separated clades that have previously been designated as CPXV-like 1 and 2 [25]. Their grouping and number is fluctuating between the current publications, but seems to be dependent on the number of analysed strains and genomic region. Dabrowski et al. identified two clades based on phylogenetic analysis of a set of highly-conserved genes within either *Poxviruses*, *Chordopoxviruses*, or *Orthopoxviruses* [26]. In an analysis of the core genome region, including intergenic regions, at least three CPXV-like clades have been described [27,42]. In contrast to that, Carrol et al. identified four different CPXV-like groups on basis of only nine CPXV isolates. However, two of these groups consisted of only a single member (Ger 91-3 and Germany 1998_2) [25].

In our analysis, including the currently broadest spectrum of 58 CPXV strains, two CPXV-like clades were identified, that mostly reflected previous groupings. However, the strains Germany 1998_2, HumLit08/1 and Ger 2010 MKY were not assigned to any certain clade, since they clustered in relative remote positions. This might indicate that the actual diversity within CPXV is much higher than reflected by previous analysis and more clades might need to be defined. Another explanation for these single branch strains might be the influence of recombination events that result in conflicting phylogenetic signals. In order to study the latter hypothesis, we addressed the phylogenetic arrangement in smaller segments of the genome and compared them in a consensus network. This type of analysis has been successfully used for detection of recombination events within other large double-stranded DNA viruses, such as human herpesvirus 1 [43]. The overall grouping into the defined clades was confirmed by this analysis, however, a certain degree of phylogenetic instability was observed between the established clades that may indicate recombination among them, especially the three strains that were not assigned into the clade system, grouped separately as a result of incompatibility between their subgenomic phylogeny. Further analysis of CPXV strain Germany 1998_2 showed that genome segments clustered with either the CPXV-like 1 or the CPXV-like 2 clade, whereas for the CPXV isolate HumLit08/1 similarities to both, the VARV-like and VACV-like clade, were observed. Recombination within OPV has, so far, been described in vivo [44,45] and in vitro [46,47,48,49], and might explain the observed mosaic genomes. However, a retrospective analysis of recombination is challenging when only limited numbers of sequences are available and the underlying phylogeny is not characterized in detail. Another limitation for this type of analysis is the fact that our alignment mainly comprises the core genome region, neglecting the more variable terminal regions, which are more frequently involved in recombination [20,50]. Recombination events among OPV might be rare, but they have to be considered in future species classification attempts, since they might violate phylogenetic analysis.

In contrast to that, the CPXV isolate Ger 2010 MKY did not display any significant recombination events with other CPXV clades. Considering the results of the recombination analysis and the remote phylogenetic position, it is very likely that Ger 2010 MKY is the prototypic member of a novel CPXV-clade, tentatively designated as “CPXV-like 3”. Ger 2010 MKY has been isolated during an outbreak in at least four captive cotton-top tamarins and has already been described as rather low pathogenic for the model species Wistar rats [30]. The unique phylogenetic position of Ger 2010 MKY was initially observed based on phylogenetic analysis of the vaccinia virus homolog F1L. Based on this separated phylogenetic position we, furthermore, investigated the phenotype of the A-type inclusion bodies (V^+^), the gene repertoire and the molecular weight of the predicted *atip* gene (150 kD), which were all typical for classical CPXV strains rather than ECTV (data not shown). Further analyses are needed in order to identify additional strains related to this novel lineage and to clarify its (rather accidental) relation to New World Monkeys. Whether this lineage represents a novel species among the OPV is, at the moment, not addressable, but could be topic of a revised nomenclature and classification of the OPVs that is urgently needed.

### 4.2. Geographic Distribution of CPXV Clades

#### 4.2.1. CPXV Situation in Germany

The origin of CPXV isolates from this study was mainly from the eastern and southern parts of Germany. This, however does not reflect a real CPXV distribution, which is better reflected by the German Animal Disease Reporting System (TSN 3.0, Germany). A total of 97 clinical cases of CPXV-infected animals were reported since 2007 in Germany (date: 20/02/2017) and all German federal states were affected, with the highest case numbers reported in Bavaria (Southwest Germany, 24/97 cases). Therefore, the spatial density of CPXV strains sampling places in this study has to be separated from the actual CPXV scenario in Germany. In addition, the bias caused by international travel and animal transport on spatio-temporal patterns has to be considered, as shown for several human CPXV cases in South and West Germany, and Northern France during 2008, 2009, and 2011 (see Figure 5). The strains from these cases were genetically uniform (VARV-like clade) and attributed to infected pet rats, which probably originated from a breeder in the Czech Republic [9,51,52,53].

Interestingly, we observed a co-circulation of CPXV-like 1 and CPXV-like 2 clades within Germany. Both clades seem to be present simultaneously in the same region as indicated by isolates Ger/2015/Cat1 (CPXV-like 2) and Ger/2015/Cat3 (CPXV-like 1). These CPXV isolates were derived from infected cats in the autumn of 2015, but are genetically different, as determined by phylogenetic analysis. Since CPXV infections in cats are believed to occur due to transmission from wild rodents during hunting, both isolates may represent adaptations to different species of small mammals that are present in the same area. In contrast, all strains isolated from alpaca-associated cases clustered solely into the CPXV-like 1 clade, although they originated from different parts of Eastern Germany. Whether these New World Camelids are more susceptible to the CPXV-like 1 clade, the spectrum of wild rodents in their direct contact is limited or the CPXV-like 1 clade is more abundant needs to be addressed in further studies. This is especially interesting since the number of New World Camelids held as livestock is growing in Germany and CPXV infections were reported repeatedly [54].

#### 4.2.2. CPXV Situation in Europe

All defined CPXV clades are present in Europe. Until now, in Great Britain only CPXV strains from the CPXV-like 2 clade were found, and a single Norwegian isolate also belongs to the CPXV-like 2 clade. Whether this clade is predominantly found in northern parts of Europe could only be confirmed by analysing more CPXV strains from these regions. In addition, the VACV-clade was only detected in the Eastern part of Europe, as well as in Austria. Again, a lack of appropriate numbers of isolates may account for that phenomenon. As stated above, central Europe seems to be a melting pot with co-circulation of CPXV-like 1, CPXV-like 2, as well as VARV-like clades. Novel zoonotic CPXV-like clades or OPV species might be present in other parts of Europe and Western Eurasia, as currently shown for the Akhmeta virus, which has been isolated from humans and cows in Georgia [55].

## 5. Conclusions

Over the last years, an increasing number of CPXV infections of humans and animals have been reported in Europe but still little is known about the genetic diversity and geographic distribution of the different CPXV clades. Here, we could show, that phylogenetic analyses based on full-length genome sequences allows a robust classification and differentiation of OPV species, as wells as different CPXV clades. Furthermore, the CPXV strain Ger 2010 MKY was identified as phylogenetically different from any other described CPXV strain and might represent the first member of a novel CPXV clade. It also indicates, that the diversity within the existing CPXV is currently underestimated and novel lineages or recombinants might emerge in geographic areas with co-circulating clades. This is of special interest, since CPXV possess a zoonotic potential and is currently considered as growing human health threat, and new CPXV strains might pose even higher risks concerning the transmission to and adaption within the human host. In the future, the identification and characterization of further CPXV strains from European countries are necessary to confirm the current phylogenetic picture and to address the question of a common ancestor of all OPV species.

## Figures and Tables

**Table 1:** Summary of 20 cowpox virus (CPXV) strains that have been isolated in Germany.

Strain	Sampling Place	Year	Host	Clinical Description
Ger/2007/Vole	Rottweil	2007	Common vole	No clinical signs
Ger/2010/Alpaca	Oberwiesenthal	2010	Alpaca	Diseased
Ger 2010 MKY	Bad Liebenstein	2010	Cotton-top tamarin	Fatal generalization
Ger/2010/Cat	Nordhausen	2010	Cat	Fatal generalization
Ger/2010/Raccoon	Ellrich	2010	Raccoon	Fatal generalization
Ger/2010/Rat	Hannover	2010	Rat	Diseased
Ger/2012/Alpaca	Rositz	2012	Alpaca	Fatal generalization
Ger/2013/Alpaca	Zernitz	2013	Alpaca	Fatal generalization
Ger/2014/Cat1	Bleckede	2014	Cat	Fatal generalization
Ger/2014/Cat2	Nordhausen	2014	Cat	Fatal generalization
Ger/2014/Human	Freiburg	2014	Human	Local lesions
Ger/2015/Cat1	Vogtlandkreis ^1^	2015	Cat	Fatal generalization
Ger/2015/Cat2	Rostock	2015	Cat	Local lesions
Ger/2015/Cat3	Vogtlandkreis ^1^	2015	Cat	Fatal generalization
Ger/2015/Cat4	Hengelbach	2015	Cat	Local lesions
Ger/2015/Prairie-dog	Dresden	2015	Prairie dog	Local lesions
Ger/2015/Human1	Leipzig	2015	Human	Cervical local lesion
Ger/2015/Human2	Leipzig	2015	Human	Local lesion
Ger/2017/Alpaca1	Brand-Erbisdorf	2017	Alpaca	Fatal generalization
Ger/2017/Alpaca2	Merzdorf	2017	Alpaca	Fatal generalization

^1^ Rural district.

## Supplementary Materials

The following are available online at www.mdpi.com/199-4915/9/6/142/s1, Table S1: Sixty-three full-length OPV sequences, included in the phylogenetic analysis; Table S2: Sampling coordinates of 58 CPXV strains; Figure S1: Phylogeny of *Orthopoxviruses*, rooted in New-World species.

## References

[1] (2015). International Committee on Taxonomy of Viruses (ICTV). Virus Taxonomy: 2015 Release.

[2] Sanchez-Sampedro L., Perdiguero B., Mejias-Perez E., Garcia-Arriaza J., Di Pilato M., Esteban M. (2015). The evolution of poxvirus vaccines. Viruses.

[3] Downie A.W. (1939). A study of the lesions produced experimentally by cowpox virus. J. Pathol. Bacteriol..

[4] Archard L.C., Mackett M. (1979). Restriction endonuclease analysis of red cowpox virus and its white pock variant. J. Gen. Virol..

[5] Chantrey J., Meyer H., Baxby D., Begon M., Bown K.J., Hazel S.M., Jones T., Montgomery W.I., Bennett M. (1999). Cowpox: Reservoir hosts and geographic range. Epidemiol. Infect..

[6] McFadden G. (2005). Poxvirus tropism. Nat. Rev. Microbiol..

[7] Vorou R.M., Papavassiliou V.G., Pierroutsakos I.N. (2008). Cowpox virus infection: An emerging health threat. Curr. Opin. Infect. Dis..

[8] Vogel S., Sardy M., Glos K., Korting H.C., Ruzicka T., Wollenberg A. (2012). The munich outbreak of cutaneous cowpox infection: Transmission by infected pet rats. Acta Derm-Venereol..

[9] Becker C., Kurth A., Hessler F., Kramp H., Gokel M., Hoffmann R., Kuczka A., Nitsche A. (2009). Cowpox virus infection in pet rat owners: Not always immediately recognized. Dtsch. Arztebl. Int..

[10] Bonnekoh B., Falk K., Reckling K.F., Kenklies S., Nitsche A., Ghebremedhin B., Pokrywka A., Franke I., Thriene B., Konig W. (2008). Cowpox infection transmitted from a domestic cat. J. Dtsch. Dermatol. Ges..

[11] Switaj K., Kajfasz P., Kurth A., Nitsche A. (2015). Cowpox after a cat scratch—Case report from poland. Ann. Agric. Environ. Med..

[12] Hemmer C.J., Littmann M., Lobermann M., Meyer H., Petschaelis A., Reisinger E.C. (2010). Human cowpox virus infection acquired from a circus elephant in Germany. Int. J. Infect. Dis..

[13] Kurth A., Wibbelt G., Gerber H.P., Petschaelis A., Pauli G., Nitsche A. (2008). Rat-to-elephant-to-human transmission of cowpox virus. Emerg. Infect. Dis..

[14] Kurth A., Straube M., Kuczka A., Dunsche A.J., Meyer H., Nitsche A. (2009). Cowpox virus outbreak in banded mongooses (*Mungos mungo*) and jaguarundis (*Herpailurus yagouaroundi*) with a time-delayed infection to humans. PLoS ONE.

[15] Fassbender P., Zange S., Ibrahim S., Zoeller G., Herbstreit F., Meyer H. (2016). Generalized cowpox virus infection in a patient with HIV, Germany, 2012. Emerg. Infect. Dis..

[16] Kinnunen P.M., Holopainen J.M., Hemmila H., Piiparinen H., Sironen T., Kivela T., Virtanen J., Niemimaa J., Nikkari S., Jarvinen A. (2015). Severe ocular cowpox in a human, Finland. Emerg. Infect. Dis..

[17] Czerny C.P., Eis-Hubinger A.M., Mayr A., Schneweis K.E., Pfeiff B. (1991). Animal poxviruses transmitted from cat to man: Current event with lethal end. Zentralbl Veterinarmed Reihe B.

[18] Haase O., Moser A., Rose C., Kurth A., Zillikens D., Schmidt E. (2011). Generalized cowpox infection in a patient with darier disease. Br. J. Dermatol..

[19] Esposito J.J., Knight J.C. (1985). *Orthopoxvirus* DNA: A comparison of restriction profiles and maps. Virology.

[20] Gubser C., Hue S., Kellam P., Smith G.L. (2004). Poxvirus genomes: A phylogenetic analysis. J. Gen. Virol..

[21] Moss B., Knipe D.M., Howley P.M. (2001). Poxviridae: The viruses and their replication. Field's Virology.

[22] Patel D.D., Pickup D.J., Joklik W.K. (1986). Isolation of cowpox virus a-type inclusions and characterization of their major protein component. Virology.

[23] Pfeffer M., Meyer H., Mercer A.A., Schmidt A., Weber O. (2007). Poxvirus diagnostics. Poxviruses.

[24] Kurth A., Nitsche A. (2007). Fast and reliable diagnostic methods for the detection of human poxvirus infections. Future Virol..

[25] Carroll D.S., Emerson G.L., Li Y., Sammons S., Olson V., Frace M., Nakazawa Y., Czerny C.P., Tryland M., Kolodziejek J. (2011). Chasing jenner’s vaccine: Revisiting cowpox virus classification. PLoS ONE.

[26] Dabrowski P.W., Radonic A., Kurth A., Nitsche A. (2013). Genome-wide comparison of cowpox viruses reveals a new clade related to variola virus. PLoS ONE.

[27] Hoffmann D., Franke A., Jenckel M., Tamosiunaite A., Schluckebier J., Granzow H., Hoffmann B., Fischer S., Ulrich R.G., Höper D. (2015). Out of the reservoir: Phenotypic and genotypic characterization of a novel cowpox virus isolated from a common vole. J. Virol..

[28] Nitsche A., Ellerbrok H., Pauli G. (2004). Detection of orthopoxvirus DNA by real-time PCR and identification of variola virus DNA by melting analysis. J. Clin. Microbiol..

[29] Miernik B., Casetti F., Panning M., Huzly D., Meyer H., Technau-Hafsi K. (2017). Multilocular facial necrosis in a young boy: A quiz. Acta Derm. Venereol..

[30] Kalthoff D., Bock W.I., Huhn F., Beer M., Hoffmann B. (2014). Fatal cowpox virus infection in cotton-top tamarins (*Saguinus oedipus*) in Germany. Vector Borne Zoonotic Dis..

[31] Katoh K., Misawa K., Kuma K., Miyata T. (2002). MAFFT: A novel method for rapid multiple sequence alignment based on fast Fourier transform. Nucleic Acids Res..

[32] Kearse M., Moir R., Wilson A., Stones-Havas S., Cheung M., Sturrock S., Buxton S., Cooper A., Markowitz S., Duran C. (2012). Geneious basic: An integrated and extendable desktop software platform for the organization and analysis of sequence data. Bioinformatics.

[33] Castresana J. (2000). Selection of conserved blocks from multiple alignments for their use in phylogenetic analysis. Mol. Biol. Evol..

[34] Nguyen L.T., Schmidt H.A., von Haeseler A., Minh B.Q. (2015). IQ-TREE: A fast and effective stochastic algorithm for estimating maximum-likelihood phylogenies. Mol. Biol. Evol..

[35] Minh B.Q., Nguyen M.A., von Haeseler A. (2013). Ultrafast approximation for phylogenetic bootstrap. Mol. Biol. Evol..

[36] Kumar S., Stecher G., Tamura K. (2016). MEGA7: Molecular evolutionary genetics analysis version 7.0 for bigger datasets. Mol. Biol. Evol..

[37] Holland B., Moulton V. Consensus networks: A method for visualising incompatibilities in collections of trees. Proceedings of the 3rd International Workshop Algorithms Bioinformatics (WABI).

[38] Huson D.H., Bryant D. (2006). Application of phylogenetic networks in evolutionary studies. Mol. Biol. Evol..

[39] Martin D.P., Posada D., Crandall K.A., Williamson C. (2005). A modified bootscan algorithm for automated identification of recombinant sequences and recombination breakpoints. AIDS Res. Hum. Retrovir..

[40] Martin D.P., Murrell B., Golden M., Khoosal A., Muhire B. (2015). RDP4: Detection and analysis of recombination patterns in virus genomes. Virus Evol..

[41] Essbauer S., Pfeffer M., Meyer H. (2010). Zoonotic poxviruses. Vet. Microbiol..

[42] Franke A., Kershaw O., Jenckel M., König L., Beer M., Hoffmann B., Hoffmann D. (2016). Fatal cowpox virus infection in an aborted foal. Vector Borne Zoonotic Dis..

[43] Kolb A.W., Ane C., Brandt C.R. (2013). Using HSV-1 genome phylogenetics to track past human migrations. PLoS ONE.

[44] Gershon P.D., Kitching R.P., Hammond J.M., Black D.N. (1989). Poxvirus genetic recombination during natural virus transmission. J. Gen. Virol..

[45] Okeke M.I., Hansen H., Traavik T. (2012). A naturally occurring cowpox virus with an ectromelia virus A-type inclusion protein gene displays atypical A-type inclusions. Infect. Genet. Evol..

[46] Hansen H., Okeke M.I., Nilssen O., Traavik T. (2004). Recombinant viruses obtained from co-infection in vitro with a live vaccinia-vectored influenza vaccine and a naturally occurring cowpox virus display different plaque phenotypes and loss of the transgene. Vaccine.

[47] Chernos V.I., Antonova T.P., Senkevich T.G. (1985). Recombinants between vaccinia and ectromelia viruses bearing the specific pathogenicity markers of both parents. J. Gen. Virol..

[48] Ball L.A. (1987). High-frequency homologous recombination in vaccinia virus DNA. J. Virol..

[49] Fathi Z., Dyster L.M., Seto J., Condit R.C., Niles E.G. (1991). Intragenic and intergenic recombination between temperature-sensitive mutants of vaccinia virus. J. Gen. Virol..

[50] Esposito J.J., Sammons S.A., Frace A.M., Osborne J.D., Olsen-Rasmussen M., Zhang M., Govil D., Damon I.K., Kline R., Laker M. (2006). Genome sequence diversity and clues to the evolution of variola (smallpox) virus. Science.

[51] Ninove L., Domart Y., Vervel C., Voinot C., Salez N., Raoult D., Meyer H., Capek I., Zandotti C., Charrel R.N. (2009). Cowpox virus transmission from pet rats to humans, France. Emerg. Infect. Dis..

[52] Campe H., Zimmermann P., Glos K., Bayer M., Bergemann H., Dreweck C., Graf P., Weber B.K., Meyer H., Buttner M. (2009). Cowpox virus transmission from pet rats to humans, Germany. Emerg. Infect. Dis..

[53] Ducournau C., Ferrier-Rembert A., Ferraris O., Joffre A., Favier A.L., Flusin O., Van Cauteren D., Kecir K., Auburtin B., Vedy S. (2013). Concomitant human infections with 2 cowpox virus strains in related cases, France, 2011. Emerg. Infect. Dis..

[54] Goerigk D., Theuß T., Pfeffer M., Konrath A., Kalthoff D., Woll D., Vahlenkamp T.W., Beer M., Starke A. (2014). Kuhpockenvirusinfektion bei einem alpaka (*Vicugna pacos*)—Klinische Symptomatik, Diagnostik und pathologische befunde. Tierärztliche Praxis Großtiere.

[55] Vora N.M., Li Y., Geleishvili M., Emerson G.L., Khmaladze E., Maghlakelidze G., Navdarashvili A., Zakhashvili K., Kokhreidze M., Endeladze M. (2015). Human infection with a zoonotic orthopoxvirus in the country of georgia. N. Engl. J. Med..

