# Supplementary material for: Classification of Cowpox Viruses into Several Distinct Clades and Identification of a Novel Lineage
Source: Viruses. 2017 Jun 10;9(6):142. doi: 10.3390/v9060142 (PMC5490819; doi:10.3390/v9060142)
Supplement: Supplementary file 1 [file viruses-09-00142-s001.zip › Supplement_revisionDH.pdf]

Supplementary Information for

Franke *et al.*: Classification of cowpox viruses into several distinct clades and identification of a novel lineage.

TABLES

Table S1: 63 full-length OPV sequences, included in the phylogenetic analysis

Species	Strain name	Collection year	Country	Host	Accession
Camelpox virus	0408151v	n.a.	n.a.	n.a.	KP768318
	CMS	1970	Iran	Dromedary	AY009089
	M-96 from Kazakhstan	1996	Kazakhstan	Dromedary	AF438165
Cowpox virus	Amadeus 2015	2015	Germany	Horse	LN879483
	Austria 1999	1999	Austria	Cat	HQ407377
	BeaBer04/1	2004	Germany	Beaver	KC813491
	Brighton Red	1939	UK	Human	AF482758
	CatBer07/1	2007	Germany	Cat	KC813502
	CatPot07/1	2007	Germany	Cat	KC813506
	EleGri07/1	2007	Germany	Elephant	KC813507
	Finland_2000_MAN	2000	Finland	Human	HQ420893
	FM2292	2011	Germany	Vole	LN864566
	France_2001_Nancy	2001	France	Human	HQ420894
	Germany 91-3	1991	Germany	Human	DQ437593
	Germany_1980_EP4	1980	Germany	Elephant	HQ420895
	Germany_1990_2	1990	Germany	Human	HQ420896
	Germany_1998_2	1998	Germany	Human	HQ420897
	Germany_2002_MKY	2002	Germany	Marmoset	HQ420898
	GRI-90	1990	Russia	Human	X94355
	HumAac09/1	2009	Germany	Human	KC813508
	HumBer07/1	2007	Germany	Human	KC813509
	HumGra07/1	2007	Germany	Human	KC813510
	HumGri07/1	2007	Germany	Human	KC813511
	HumKre08/1	2008	Germany	Human	KC813512
	HumLan08/1	2008	Germany	Human	KC813492
	HumLit08/1	2008	Germany	Human	KC813493
	HumLue09/1	2009	Germany	Human	KC813494
	HumMag07/1	2007	Germany	Human	KC813495
	HumPad07/1	2007	Germany	Human	KC813496
	JagKre08/1	2008	Germany	Jaguarundi	KC813497
	JagKre08/2	2008	Germany	Jaguarundi	KC813498
	Kostroma_2015	2015	Russia	Human	KY369926
	MarLei07/1	2007	Germany	Mara	KC813499

	MonKre08/4	2008	Germany	Mongoose	KC813500
	Norway_1994_MAN	1994	Norway	Human	HQ420899
	RatAac09/1	2009	Germany	Rat	KC813501
	RatGer09/1	2009	Germany	Rat	KC813503
	RatHei09/1	2009	Germany	Rat	KC813504
	RatKre08/2	2008	Germany	Rat	KC813505
	RatPox09	2009	Germany	Rat	LN864565
	UK2000_K2984	2000	United Kingdom	Cat	HQ420900
Ectromelia virus	Moscow	1947	Russia	Mouse	AF012825
	Naval	1996	USA	Mouse	KJ563295
	VR-1431	1987	China	Human	JQ410350
Monkeypox virus	Cote d'Ivoire_1971	1971	Cote d'Ivoire	Human	KP849470
	Ivory Coast 2012	2012	Cote d'Ivoire	Monkey	KJ136820
	Liberia_1970_184	1970	Liberia	Human	DQ011156
	Sudan 2005_01	2005	Sudan: Nuria	Human	KC257459
Raccoonpox virus	Raccoonpox virus 85A	1964	USA	Racoon	KU749309
	Raccoonpox virus Herman	1964	USA	Racoon	KP143769
Skunkpox virus	Skunkpox virus	1978	USA	Skunk	KU749310
Taterapox virus	Taterapox virus Dahomey 1968	1968	Benin	Gerbil	DQ437594
Vaccina virus	Copenhagen	n.a.	n.a.	n.a.	M35027
	DUKE	1970	USA	Human	DQ439815
	Horsepox virus	1976	Mongolia	Horse	DQ792504
	Rabbitpox virus	1941	Netherland	Rabbit	AY484669
	WR	n.a.	n.a.	n.a.	NC_006998
Variola virus	Congo 1970	1970	Congo	Human	DQ437583
	Germany 1958 Heidelberg	1958	Germany	Human	DQ437584
	Guinea 1969 (005)	1969	Guinea	Human	DQ441426
	United Kingdom 1952 Butler	1952	United Kingdom	Human	DQ441447
	VD21, 17th century	1654	Lithuania	Human	KY358055
Volepox virus	Volepox virus CA	1985	USA	Vole	KU749311

n.a.: not available

Table S2: Sampling coordinates of 58 CPXV strains

**Strain name**	**Clade**	**Town**	**lat**	**lon**
Amadeus	CPXV-like 1	Berlin	52.520007	13.404954
Austria	VACV-like	Texing	48.041870	15.329212
BeaBer04/1	CPXV-like 1	Berlin	52.520007	13.404954
Brighton Red	CPXV-like 2	Brighton	50.822530	-0.137163
CatBer07/1	CPXV-like 1	Berlin	52.520007	13.404954
CatPot07/1	CPXV-like 1	Potsdam	52.390569	13.064473
EleGri07/1	CPXV-like 1	Grimmen	54.108146	13.037749
Finland_2000_MAN	VACV-like	Tohmajärvi	62.225171	30.335391
FM2292	CPXV-like 2	Rutesheim	48.808150	8.945504
France_2001_Nancy	CPXV-like 2	Nancy	48.692054	6.184417
Ger/2007/Vole	CPXV-like 2	Rottweil	48.165258	8.628453
Ger/2010/Alpaca	CPXV-like 1	Oberwiesenthal	50.418622	12.969410

Ger/2010/Cat	CPXV-like 1	Nordhausen	51.499079	10.791581
Ger/2010/Racoon	CPXV-like 1	Ellrich	51.586546	10.662888
Ger/2010/Rat	CPXV-like 1	Hannover	52.375892	9.732010
Ger 2010 MKY	(CPXV-like 3)	Bad Liebenstein	50.815306	10.349743
Ger/2012/Alpaca	CPXV-like 1	Rositz	51.021516	12.373653
Ger/2013/Alpaca	CPXV-like 1	Zernitz	52.015532	12.084589
Ger/2014/Cat1	CPXV-like 1	Bleckede	53.286547	10.733486
Ger/2014/Cat2	CPXV-like 1	Nordhausen	51.499079	10.791581
Ger/2014/Human	CPXV-like 2	Freiburg	47.999008	7.842104
Ger/2015/Cat1	CPXV-like 2	Vogtlandkreis	50.489135	12.280376
Ger/2015/Cat2	CPXV-like 1	Rostock	54.092441	12.099147
Ger/2015/Cat3	CPXV-like 1	Vogtlandkreis	50.489135	12.280376
Ger/2015/Cat4	CPXV-like 1	Hengelbach	50.724078	11.110978
Ger/2015/Human1	CPXV-like 1	Leipzig	51.339695	12.373075
Ger/2015/Human2	CPXV-like 1	Leipzig	51.339695	12.373075
Ger/2015/Prairie Dog	CPXV-like 1	Dresden	51.050409	13.737262
Germany 91-3	CPXV-like 2	Munich	48.135125	11.581981
Germany 1980_EP4	CPXV-like 1	Hameln	52.108273	9.362171
Germany 1990_2	CPXV-like 2	Bonn	50.737430	7.098207
Germany 1998_2	n.a.	Eckental	49.574179	11.201352
Germany_2002_MKY	CPXV-like 1	Göttingen	51.541280	9.915804
GRI-90	VACV-like	Moscow	55.755826	37.617300
HumAac09/1	VARV-like	Aachen	50.775346	6.083887
HumBer07/1	CPXV-like 1	Berlin	52.520007	13.404954
HumGra07/1	VARV-like	Graz	47.070714	15.439504
HumGri07/1	CPXV-like 1	Grimmen	54.108146	13.037749
HumKre08/1	VARV-like	Krefeld	51.338761	6.585342
HumLan08/1	CPXV-like 1	Landau	49.198886	8.118562
HumLit08/1	n.a.	Vilnius	54.687156	25.279651
HumLue09/1	CPXV-like 2	Lübeck	53.865467	10.686559
HumMag07/1	CPXV-like 1	Magdeburg	52.120533	11.627624
HumPad07/1	CPXV-like 1	Paderborn	51.718921	8.757509
JagKre08/1	CPXV-like 1	Krefeld	51.338761	6.585342
JagKre08/2	CPXV-like 1	Krefeld	51.338761	6.585342
MarLei07/1	CPXV-like 2	Leipzig	51.339695	12.373075
MonKre08/4	CPXV-like 1	Krefeld	51.338761	6.585342
Norway_1994_MAN	CPXV-like 2	Bergen	60.391263	5.322054
RatAac09/1	VARV-like	Aachen	50.775346	6.083887
RatGer09/1	VARV-like	Germering	48.135181	11.369089
RatHei09/1	CPXV-like 1	Heidelberg	49.398752	8.672434
RatKre08/2	VARV-like	Krefeld	51.338761	6.585342
Ratpox09	VARV-like	Munich	48.135125	11.581981
UK2000_K2984	CPXV-like 2	Bristol	51.454513	-2.587910
Kostroma_2015	VACV-like	Kostroma	57.777482	40.969893
Ger/2017/Alpaca1	CPXV-like 1	Brand-Erbisdorf	50.866688	13.323660
Ger/2017/Alpaca2	CPXV-like 1	Merzdorf	51.403338	13.538144

n.a.: not applicable

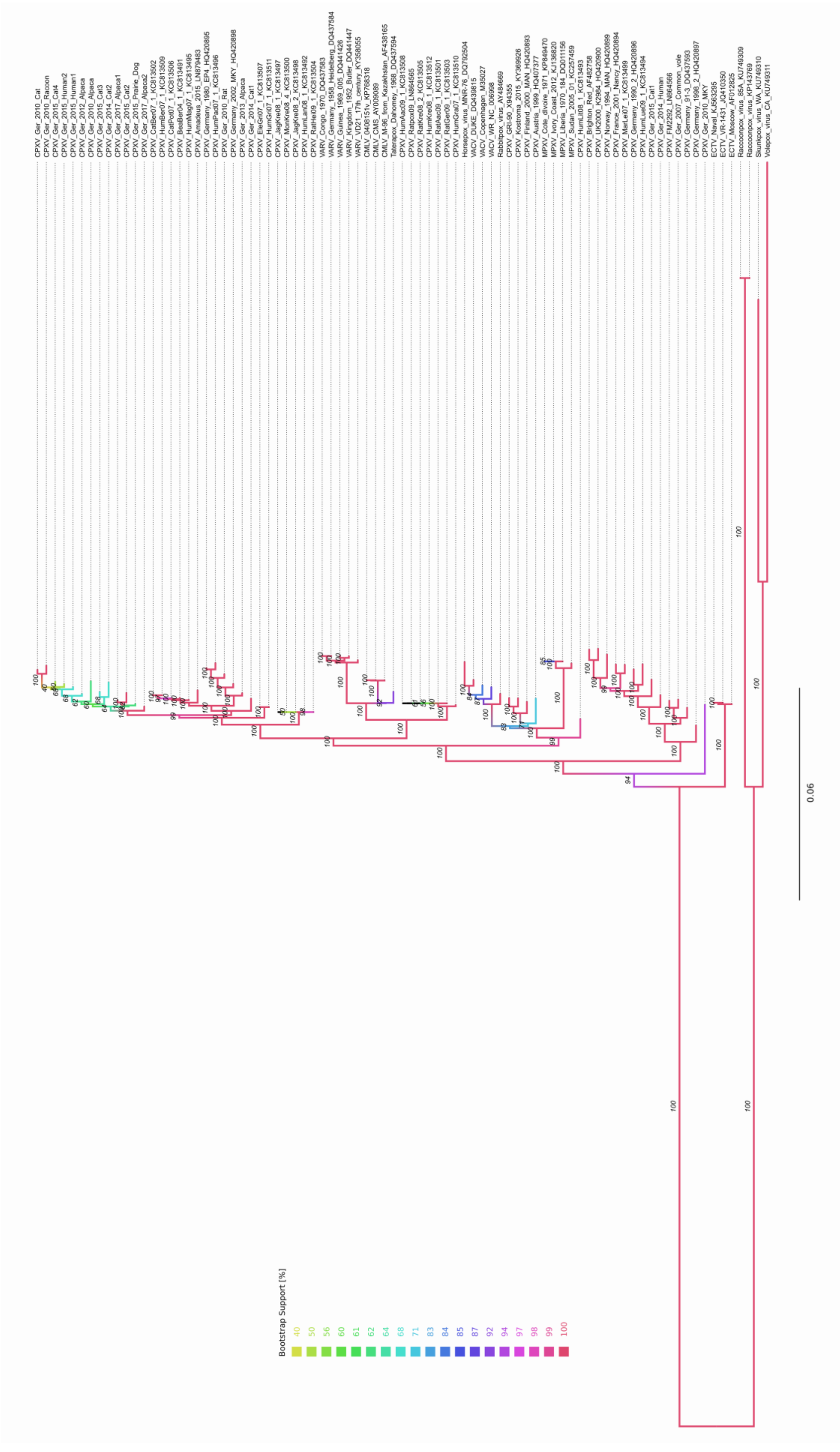

Figure S1: Phylogeny of Orthopoxviruses, rooted at New-World species. Scale represent substitutions per position. Statistic support by 100.000 ultrafast bootstraps is indicated for each branch by colour indication in addition to individual % value.
