# Supplementary figures and images for: Classification of Cowpox Viruses into Several Distinct Clades and Identification of a Novel Lineage
Source: Viruses. 2017 Jun 10;9(6):142. doi: 10.3390/v9060142 (PMC5490819; doi:10.3390/v9060142)

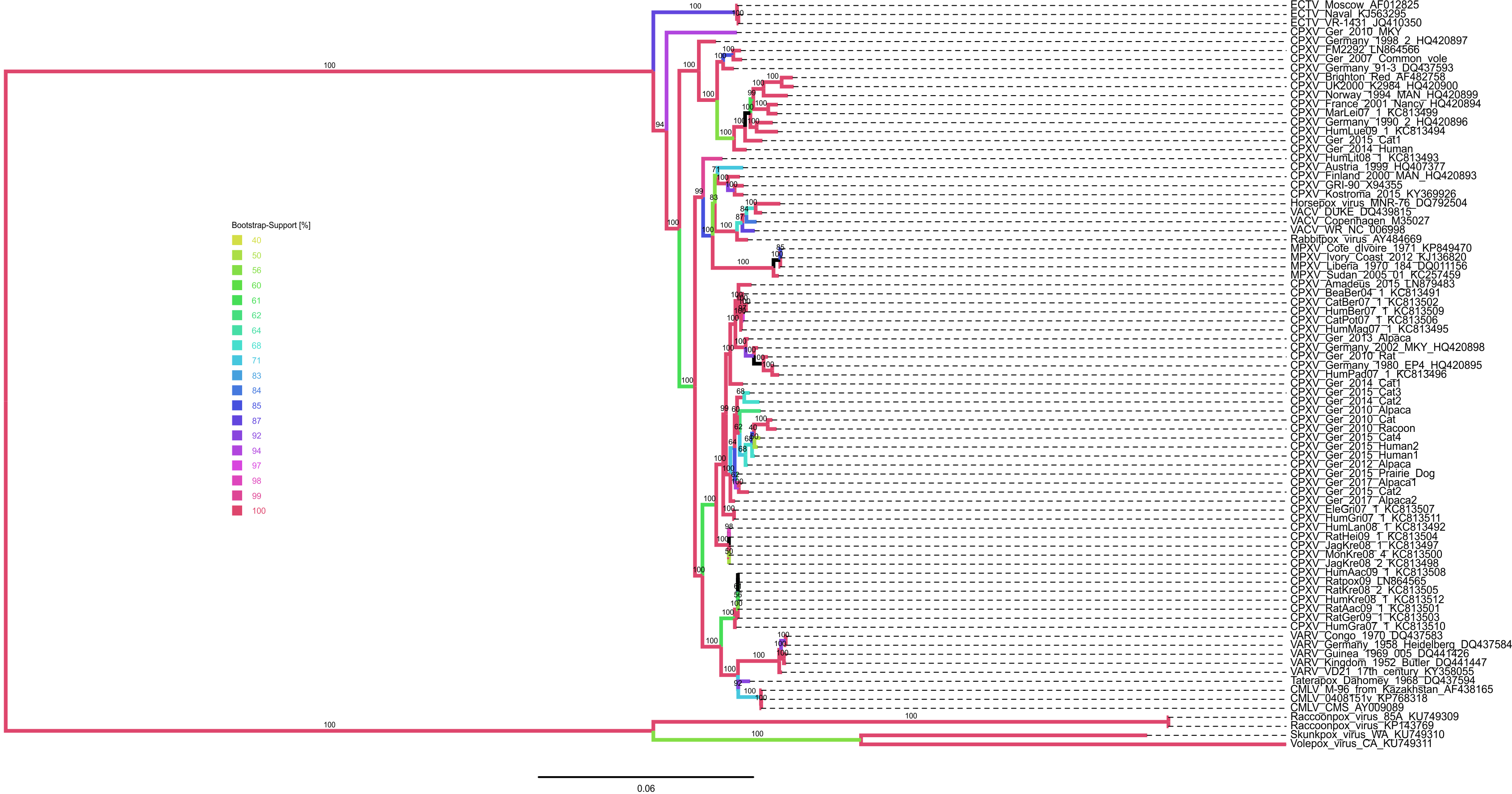

Supplement: Supplementary file 1 [file viruses-09-00142-s001.zip › SupplementalFigure1.png]
